# NEDL2 regulates enteric nervous system and kidney development in its Nedd8 ligase activity-dependent manner

**DOI:** 10.18632/oncotarget.8951

**Published:** 2016-04-23

**Authors:** Xiao Qiu, Rongfei Wei, Yang Li, Qiong Zhu, Cong Xiong, Yuhan Chen, Yuan Zhang, Kefeng Lu, Fuchu He, Lingqiang Zhang

**Affiliations:** ^1^ School of Life Sciences, Tsinghua University, Beijing 100084, China; ^2^ State Key Laboratory of Proteomics, Beijing Proteome Research Center, Beijing Institute of Radiation Medicine, Collaborative Innovation Center for Cancer Medicine, Beijing 100850, China; ^3^ Institute of Cancer Stem Cell, Dalian Medical University, Dalian 116044, China; ^4^ Department of Experimental Pathology, Beijing Institute of Radiation Medicine, Beijing 100850, China

**Keywords:** kidney development, GDNF/Ret/Akt pathway, scaffold protein, ubiquitin ligase, Nedd8 ligase

## Abstract

The GDNF (Glial cell line-derived neurotrophic factor)/Ret/Akt signaling pathway is essential to the development of ENS (enteric nervous system) as well as kidney. We previously showed that the HECT-type E3 ligase NEDL2 (Nedd4-like ligase 2) is required for the ENS development by activating GDNF/Ret/Akt. However, the underlying mechanism remains unknown. Here we show that in addition to ENS, NEDL2 is also pivotal for kidney development since about 1/3 of *Nedl2*-deficient mice displayed postnatal unilateral or bilateral kidney hydronephrosis. Double knockout of *Nedl1* and *Nedl2* in mice leads to postnatal lethal within 2 weeks and the phenotypes resemble those of *Nedl2* single knockout mice. Surprisingly, its close member NEDL1 is dispensable for ENS and kidney function and the reason is lack of NEDL1 expression in these systems during early development. Furthermore, biochemical analysis indicated that NEDL2 appears to act like a scaffold protein to recruit SHC, Grb2, PI3K (p110 and p85), PDK1 and Akt together to promote the signaling transduction. Intriguingly, we found that NEDL2 harbours intrinsic Nedd8 ligase activity with cysteine 1341 as the core site. NEDL2 upregulates GDNF-stimulated Akt activity dependent of its Nedd8 ligase activity but not its ubiquitin ligase activity. These findings demonstrate that NEDL2 but not NEDL1 is required for ENS and kidney development in a unique Nedd8 ligase-dependent manner.

## INTRODUCTION

It has been demonstrated that GDNF/Ret signaling positively regulates both ENS and kidney development [1, 2]. Following GDNF stimulation, SHC connects Ret to the cytoplasmic Grb2-Gab1 complex; recruitment of the PI3K complex through the SH2 domain of the regulatory p85 subunit to phosphorylated tyrosine residues of Gab1 promotes the translocation of PI3K to the plasma membrane and leads to phosphatidylinositol-3,4,5-triphophosphate (PIP3) production [2, 3]. PIP3 serves as a second messenger, and one of the critical targets of PIP3 is Akt. Upon binding of PIP3, Akt translocates to the cellular membrane where it is activated by PDK1 and mTORC kinases [4, 5]. TRAF6 was reported to ubiquitinates Akt, thus promotes membrane recruitment of Akt under IGF-1 stimulation [6]. Reversely, CYLD negatively regulates Akt signaling by deubiquitinating Akt in TGFβ signaling [7]. However, the regulatory mechanism of PI3K/Akt signaling, in particularly under GDNF/Ret axis, remains not fully understood.

Through genetic analysis of knockout mice model, we recently identified the HECT domain-type ubiquitin ligase NEDL2, which belongs to the Nedd4 ligase family, as an essential positive regulator of ENS development via GDNF/Ret/Akt pathway and *Nedl2* deficiency leads to mice lethal within postnatal 2 weeks [8]. So far, NEDL2 is the sole one reported to be required for ENS development control among the whole Nedd4 ligase family which consists of nine members in mammals. This family ligase all contains the C2-WW-HECT architecture and functions as typical ubiquitin ligase [9]. Notably, the yeast ortholog of these family, Rsp5, and the mammalian member Smurf1 can also function as a Nedd8 (neural precursor cell expressed developmentally downregulated protein 8) ligase [10]. Nedd8 has the greatest similarity among the ubiquitin-like proteins and protein neddylation plays a diverse role in normal organ development as well as in tumorigenesis and neurodegeneration diseases [11–16]. However, the relationship between neddylation and ENS development has not been reported.

In this study, we established *Nedl1* knockout and *Nedl1;Nedl2* double knockout mice. Phenotype analysis indicated a specific role of NEDL2 in ENS and kidney development. We further show that NEDL2 regulates GDNF/Ret/Akt signaling in an unexpected Nedd8 ligase activity-dependent but ubiquitin ligase activity-independent manner.

## RESULTS

### Kidney development defects in *Nedl2^−/−^* mice

**Figure 1:**
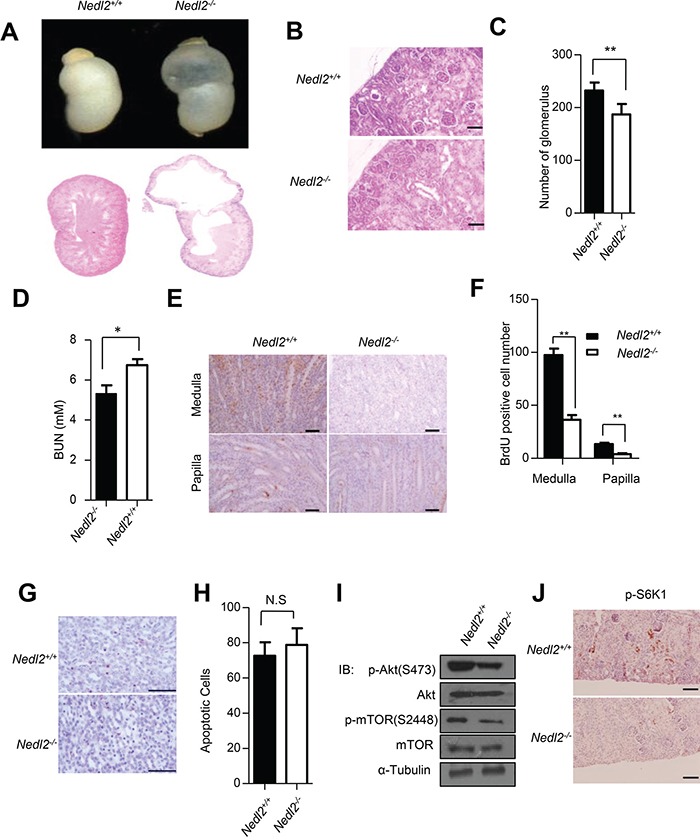
Kidney development defects in *Nedl2^−/−^* mice **A.** About 38% (5/13) of *Nedl2-*deficient newborn mice showed unilateral or bilateral hydronephrosis of distended kidneys (upper panel), HE staining of kidney sections from newborn *Nedl2^+/+^* and *Nedl2^−/−^* mutant mice showing severe developmental defect of medulla in *Nedl2^−/−^* mice (lower panel). **B-C.** The presented images (400×) were from *Nedl2^+/+^* and *Nedl2^−/−^* mutant mice of the same littermate (B), and the number of glomerular reduced in *Nedl2*-deficient mice. Statistical analysis of and Glomeruli were counted in the maximal cross-section of each P5 kidney. Five mice were used for each group (C). **D.** Significant difference of serum level of BUN between *Nedl2^+/+^* and *Nedl2^−/−^* mutant mice. (BUN, blood urea nitrogen). **E-F.** Number of BrdU-positive cells in kidney of *Nedl2^−/−^* mice was less than that of in *Nedl2^+/+^* mice, despite medulla or papilla(E). And the values are presented in (F). **G.** Apoptotic cells were identified by terminal deoxynucleotidyl-transferase-mediated dUTP nick and labeling (TUNEL) staining in kidney of *Nedl2^+/+^* mice and *Nedl2^−/−^* mice (P12). No statistical significance existed **H**. **I.** Western blot analysis of GDNF/Ret/Akt signaling pathway, fresh kidneys were dissected from P9 mice. **J.** Significant reduction of p-S6K1 level in *Nedl2^−/−^* mice kidney compared with that of *Nedl2^+/+^* mice. Scale bar: 50 μm. Values represent means ± s.d. (*P<0.05, **P<0.01).

GDNF/Ret signaling has been demonstrated to be pivotal for both kidney and ENS development [17]. We recently reported that all of the *Nedl2*-deficient mice died within 2 weeks after birth, showing low body weight. These mice showed a progressive bowel motility defect resulting from intestinal aganglionosis [8]. More careful analysis led us to find that about 38% (5/13) of *Nedl2^−/−^* mutants showed unilateral or bilateral kidneys hydronephrosis (Figure 1A upper panel). Histological analysis of these mutant kidneys showed severe dysplasia with hydronephrosis (Figure 1A lower panel). Mammalian kidney development is a complex progress. The reciprocal inductive interactions between epithelial cells and metanephric mesenchymal cells result in cell proliferation, growth, apoptosis and the the formation of kidney. The glomeruli mainly develop from epithelial cells, and the collecting ducts mainly develop from metanephric mesenchymal cells [18, 19]. Since collecting ducts system has been found defect, we compared nephron number of kidneys at postnatal day 5 (P5) and found that the number of glomeruli in the mutant kidneys reduced. Glomerular number in *Nedl2^−/−^* mutants was only 80% of that of wild-type controls (Figure 1B and 1C). Furthermore, the increased level of BUN (blood urea nitrogen) in *Nedl2^−/−^* mutants confirmed the dysplasia of kidney (Figure 1D). To more closely study the role of NEDL2 in the kidney development, we investigated whether knockout of *Nedl2* affected the kidney cell proliferation, since it has been reported that NEDL2 promotes cell proliferation [8, 20]. We labeled the proliferating cell with BrdU and found that there was a significant decrease in cellular proliferation, as evidenced by cells positive for BrdU in the mutant kidney medulla and papilla (Figure 1E and 1F). However, no statistic significance in TUNEL (terminal transferase-mediated dUTP nickend labeling)-positive cells was observed (Figure 1G and 1H). Just like in ENS, we also found that compared with wild type littermates, the GDNF/Ret/Akt pathway was downregulated in *Nedl2^−/−^* mice kidneys (Figure 1I and 1J). Collectively, the finding that the *Nedl2*-null mice show kidney hydronephrosis is in agreement with our previous study that NEDL2 is a key positive regulator of GDNF/Ret pathway [8].

### NEDL1 is not critical for survival

**Figure 2:**
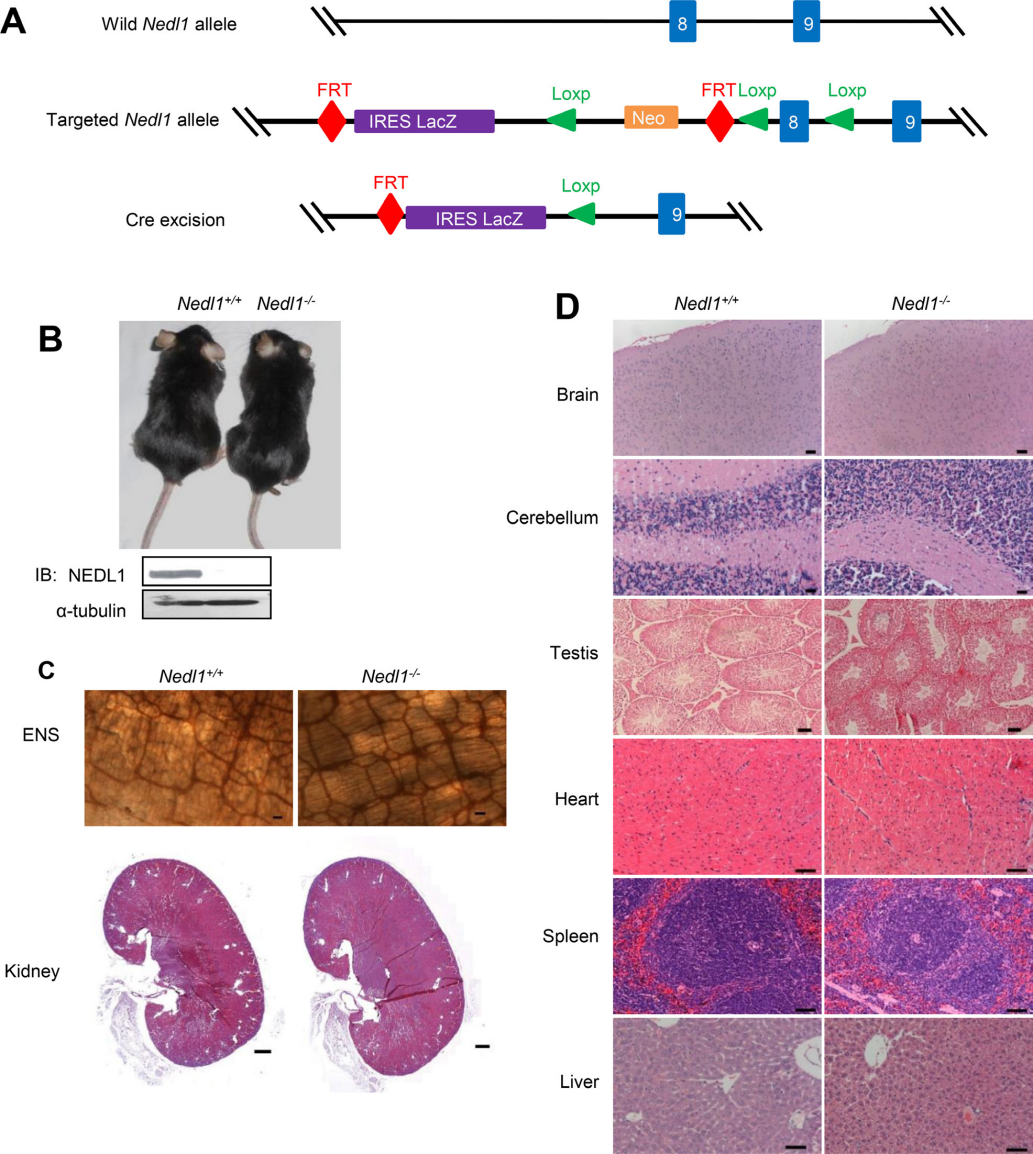
NEDL1 is not critical for mice survival **A.** Schematic diagram of the generation of *Nedl1^−/−^* mice. *Nedl1* mutant mice were generated from a C57BL/6J ES clone carrying the targeted *Nedl1* allele designed to allow Cre-mediated deletion. Mice carrying the targeted *Nedl1* allele were bred to EIIa-Cre transgenic mice to remove the *neo^r^* gene and generate *Nedl1^LacZ/+^* reporter mice in which exon 8 are deleted and replaced with an IRES-*LacZ* gene. Offspring were intercrossed to generate homozygote *Nedl1^LacZ/LacZ^* (*Nedl1^−/−^*) mice. Exons are represented by blue boxes. **B.** Photograph of 18-month-old *Nedl1^+/+^* and *Nedl1^−/−^* mice. **C.** ENS system detected by AChE-staining (upper panel) 2-month-old *Nedl1^+/+^* mice and *Nedl1^−/−^* mice (Scale bar: 50 μm) and HE staining of kidneys (lower panel) from 6-month-old *Nedl1^+/+^* mice and *Nedl1^−/−^* mice (Scale bar: 500 μm) show that there were no obvious defects in ENS and kidney. **D.** Analysis on HE staining of some tissue sections from 2-month-old *Nedl1^+/+^* mice and *Nedl1^−/−^* mice. There were no obvious defects in brain, cerebellum, testis, heart, liver, spleen and lung. Scale bar: 50 μm.

Among the nine members of mammalian Nedd4 family, NEDL1 shares the highest sequence similarity with NEDL2. We speculated that NEDL1 might have functional similarity with NEDL2 and if this is true, *Nedl1^−/−^*;*Nedl2^−/−^* double knockout mice should exhibit more severe phenotypes than *Nedl2* single knockout mice, like the case of Smurf1 and Smurf2 [21]. To test this hypothesis, we firstly used Cre-Loxp technology to generate *Nedl1*-null mice (Figure 2A). Homozygous *Nedl1^−/−^* mice were born at the expected Mendelian frequency (Supplementary Table S1). In addition, *Nedl1*-deficient mice, both male and female, were viable and fertile, and that homozygous *Nedl1^−/−^* females could raise their pups; there was no morphological difference between *Nedl1^−/−^* and wild-type littermates until 18 months of age (Figure 2B). Further analysis showed that unlike *Nedl2^−/−^* mice, there were no significant ENS or kidney dysplasias in *Nedl1^−/−^* mice (Figure 2C). To confirm that there were no histological differences in other organs of *Nedl1^−/−^*, main organs were derived from 2-months-old *Nedl1^−/−^* and *Nedl1^+/+^* mice, and histopathological analysis of those organs did not reveal any obvious defects in the homozygous mutants (Figure 2D). Thus, these data indicated that NEDL1 is not critical for mice survival.

### NEDL2, but not NEDL1, is critical for kidney and intestine development

**Figure 3:**
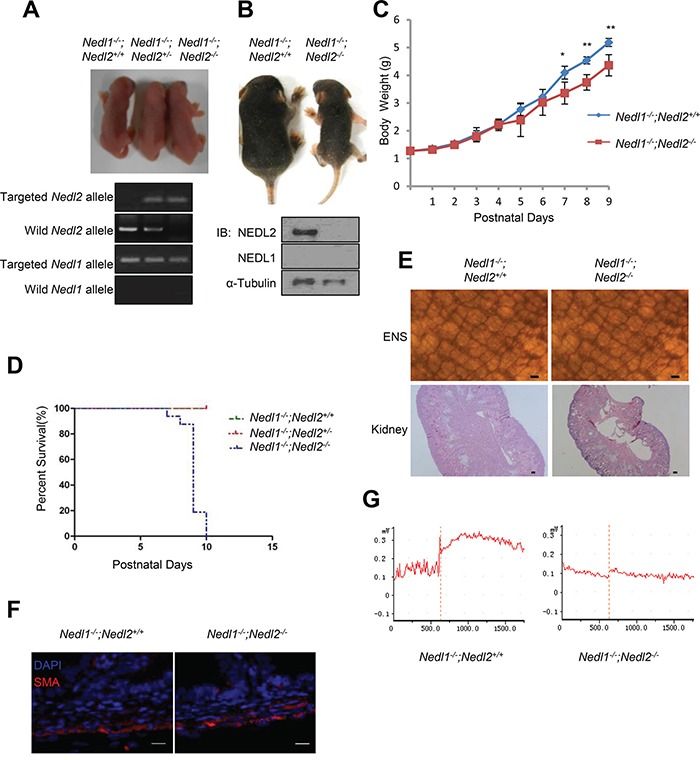
The phenotype of *Nedl1^−/−^;Nedl2^−/−^* mice **A.** Gross morphology of postnatal day 0 (P0) pups. **B.** Photograph of 9-days-old *Nedl1^−/−^*;Nedl2*^−/−^* mice littermate mice. **C.** *Nedl1^−/−^*;Nedl2*^−/−^* mice gain less body weight than *Nedl1^−/−^*;Nedl2*^+/+^* littermates despite male or female. *Nedl1^−/−^*;Nedl2*^+/+^*, (n=8); *Nedl1^−/−^*;Nedl2*^−/−^*: (n=5-8), for all time points measured. **D.** Postnatal lethality phenotype in *Nedl1^−/−^*;Nedl2*^−/−^* mice. Survival curve for *Nedl1^−/−^*;Nedl2*^+/+^*(*n*=42), *Nedl1^−/−^*;Nedl2*^+/−^* (*n*=21) and *Nedl2^−/−^* mice (*n*=16) that overcome neonatal death. **E.** ENS system detected by AChE-staining (upper panel) and HE staining of kidneys (lower panel) from 9-day-old *Nedl1^−/−^*;*Nedl2^+/+^* mice and *Nedl1^−/−^*; *Nedl2^−/−^* mice showed that there were obvious defects in ENS and kidney. Scale bar: 50 μm. **F.** Immunohistochemistry using anti-smooth muscle α-actin (SMA) antibody showed normal thickness of the smooth muscle layer in *Nedl1^−/−^*;Nedl2*^+/+^* mice and *Nedl1^−/−^*;Nedl2*^−/−^* mice. Scale bar: 20 μm. **G.** Recording of intestine contraction force in response to 10 mM carbachol (CCh). Time periods are indicated during CCh treatment.

The above data suggested that NEDL1 was not required for the ENS and kidney development and its deficiency did not result in mice death. To precisely clarify whether NEDL1 has functional redundancy with NEDL2, we further established double mutant *Nedl1^−/−^; *Nedl2*^−/−^* mice. Firstly, we generated *Nedl1^−/−^*;Nedl2*^+/−^* mice, these mice (which appeared phenotypically normal and fertile) were then intercrossed to generate double homozygous *Nedl1^−/−^*;*Nedl2^−/−^* embryos. *Nedl1^−/−^*;*Nedl2^−/−^* double homozygous mutant were born at the expected frequency (Supplementary Table S2) and *Nedl1^−/−^*;*Nedl2^−/−^* mice did not appear to differ phenotypically from *Nedl1^−/−^*;*Nedl2^+/−^* and *Nedl1^−/−^*;*Nedl2^+/+^* littermates at birth (Figure 3A), however, all *Nedl1^−/−^*;*Nedl2^−/−^* mice died within 2 weeks after birth with low body weight (Figures 3B-3D). The double homozygous *Nedl1^−/−^*;*Nedl2^−/−^* mice exhibited the defects characteristic of *Nedl2* single mutant we have been reported [8]. So we next tested whether there were dysplasias in the ENS and kidneys in *Nedl1^−/−^*;*Nedl2^−/−^* mice. Whole-mount AChE staining showed that number of ENS cells was decreased in the *Nedl1^−/−^*;*Nedl2^−/−^* mice and HE staining showed hydronephrosis in kidneys (Figure 3E). Just like *Nedl2* single mutant, the thickness of the circular muscle or the longitudinal muscle in *Nedl1^−/−^*; *Nedl2^−/−^* mice was indistinguishable change (Figure 3F). Furthermore, we tested the intestine contractile responses to CCh (carbachol) in *Nedl1^−/−^*;*Nedl2^−/−^* mice and control littermates, the maximum contraction force was significantly reduced in the *Nedl1^−/−^*;*Nedl2^−/−^* compared with *Nedl1^−/−^*;*Nedl2^+/+^* littermates (Figure 3G). In addition, no obvious defects were observed in the organs that showed in our histological examinations (Supplementary Figure S1).

**Figure 4:**
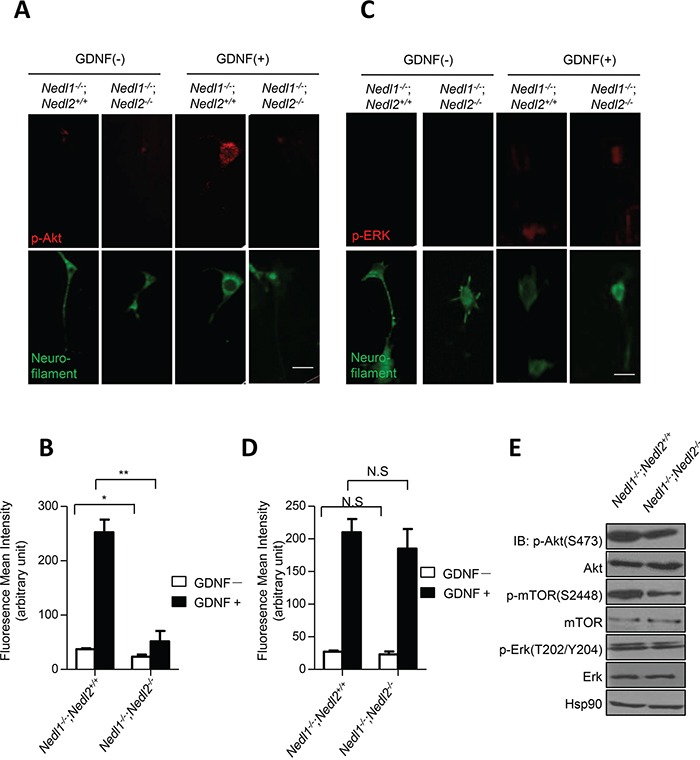
Suppression of GDNF/Akt signaling in *Nedl1^−/−^;Nedl2^−/−^* mice **A**–**B.** Akt phosphorylation in cultured enteric neurons derived from P9 intestine after GDNF stimulation. Cells with or without 30 min treatment of GDNF stained with anti-pAkt antibody. Scale bar: 20 μm. **C-D.** Erk phosphorylation in cultured enteric neurons derived from P9 colon after GDNF stimulation. Cells with or without 5 min treatment of GDNF stained with anti-pErk antibody. Scale bar: 20 μm. **E.** Western blot analysis of GDNF/Ret/Akt signaling pathway, fresh tissues are dissected from P9 mice. Values represent means ± s.d. (*P<0.05, **P<0.01).

Our previous studies have demonstrated inactivation of GDNF/Ret/Akt signaling in *Nedl2^−/−^* mutant mice [8]. Thus, changes in GDNF/Ret/Akt signaling were examined in cultured *Nedl1^−/−^*;*Nedl2^−/−^* and *Nedl1^−/−^*;*Nedl2^+/+^* enteric neurons. As expected, after GDNF stimulation, neurons from *NedlL1^−/−^*;*Nedl2^−/−^* mice exhibited reduced levels of pAkt (Figure 4A and 4B), but not of p-Erk, compared with *Nedl1^−/−^*;*Nedl2^+/+^* neurons (Figures 4C and 4D). We also analyzed the changes of GDNF/Ret/Akt signaling in *Nedl1^−/−^*;*Nedl2^−/−^* and *Nedl1^−/−^*;*Nedl2^+/+^* mice intestine tissues with immunoblotting analysis. As shown, inactivation of GDNF/Ret signaling in *Nedl1^−/−^*;*Nedl2^−/−^* mice were confirmed (Figure 4E).

**Figure 5:**
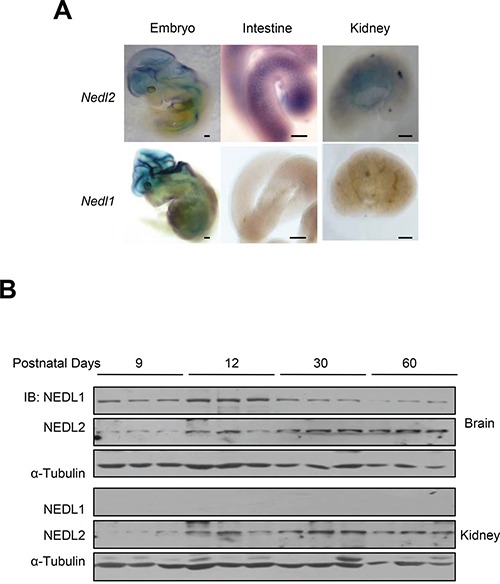
Different expression patterns of NEDL1 and NEDL2 **A.** Expression of *Nedl2* (upper) and *Nedl1* (lower) mRNA in E12.5 embryo, E18.5 intestine and kidney as detected by *in situ hybridization* indicated that NEDL2 is expressed differently with NEDL1. Scale bar: 400 μm **B.** Immunoblotting analysis of NEDL1 and NEDL2 expression in mouse kidneys and brains. Fresh tissue samples are dissected from different ages of mice, which indicated as shown, the protein level of NEDL1 and NEDL2 is tested by immunoblotting. Every stage is tested with 3 mice.

Why NEDL1 and NEDL2 harbours similar protein sequence but *Nedl1^−/−^* and *Nedl2^−/−^* mice showed so different phenotypes? To address this issue, we investigated the expression pattern of NEDL1 and NEDL2, and surprisingly found that although both were expressed in central nervous system (CNS), only NEDL2 was expressed in the ENS system and kidney during the early stage of development (Figure 5A and 5B). This difference can explain why *Nedl1* deletion did not cause significant defects in kidney and ENS. Taken together, these findings indicated that comparing with NEDL1, NEDL2 plays a more significant role in the early development of mice and strengthen the notion that NEDL2 is a pivotal positive regulator of GDNF/Ret/Akt pathway.

### NEDL2 acts as a scaffold protein to promote GDNF-stimulated Akt activation

**Figure 6:**
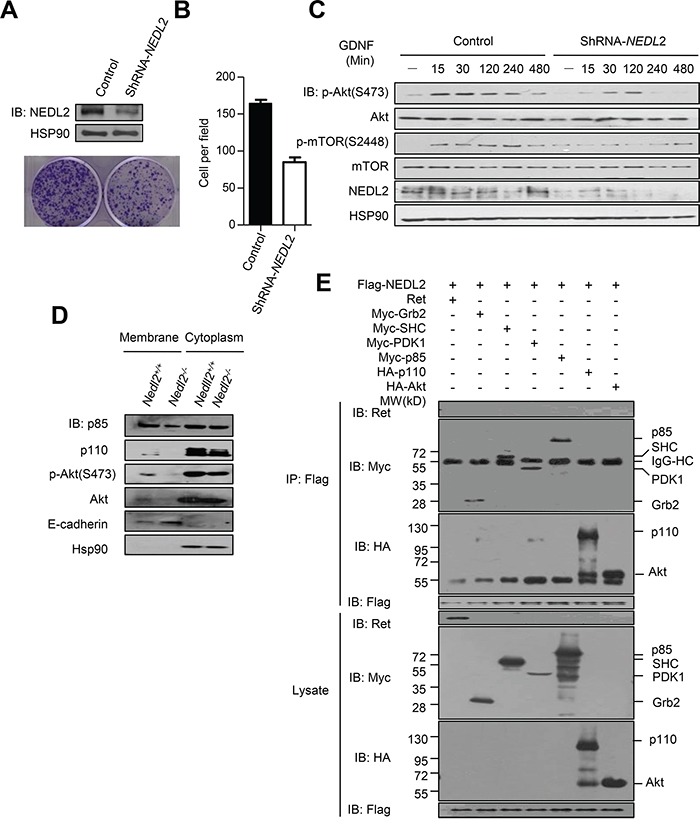
NEDL2 acts as a scaffold protein to promote GDNF-stimulated Akt activation **A-C.** NEDL2 is a positive regulator of PI3K-Akt pathway in MCF7 cells. Stable knockdown of *NEDL2* expression in MCF7 cells using specific shRNA targeting *NEDL2* mRNA. Clone formation assay(A) and rate (B) of MCF7 cells with stably expressed control ShRNA or *NEDL2*-ShRNA. Cells were serum-starved overnight and treated with GDNF (40 ng/mL) for indicated time and then collected for immunoblotting analysis with the indicated antibodies(C). **D.** NEDL2 positively regulates the plasma translocation of p85, p110 and Akt. Protein samples from *Nedl2^+/+^* and *Nedl2^−/−^* mice Intestine tissues were separated into membrane fractions and non-membrane fractions. The membrane fractions and non-membrane fractions were collected for immunoblotting analysis with the indicated antibodies. **E.** NEDL2 interact with SHC, Grb2, PDK1, p85, p110 and Akt. HEK293T cells were transfected with Flag-NEDL2 and Ret/Myc-SHC/Myc-Grb2/Myc-PDK1/Myc-p85/HA-p110/HA-Akt expression plasmids. Forty-eight hours after transfection, cell lysates were prepared and immunoprecipitated with anti-Flag antibody. The immunoprecipitates were analyzed by immunoblotting with the indicated antibodies.

In order to investigate whether NEDL2 plays a universal role in regulating GDNF/Ret/Akt pathway, we use shRNA to stable knockdown of *NEDL2* gene expression in MCF7 cells. In clone formation assay, we found that knockdown of *NEDL2* gene expression led to the reduction of cell proliferation (Figure 6A and 6B). In addition, stable knockdown of *NEDL2* resulted in low level activation of GDNF/Ret/Akt pathway when stimulated with GDNF (Figure 6C).

**Figure 7:**
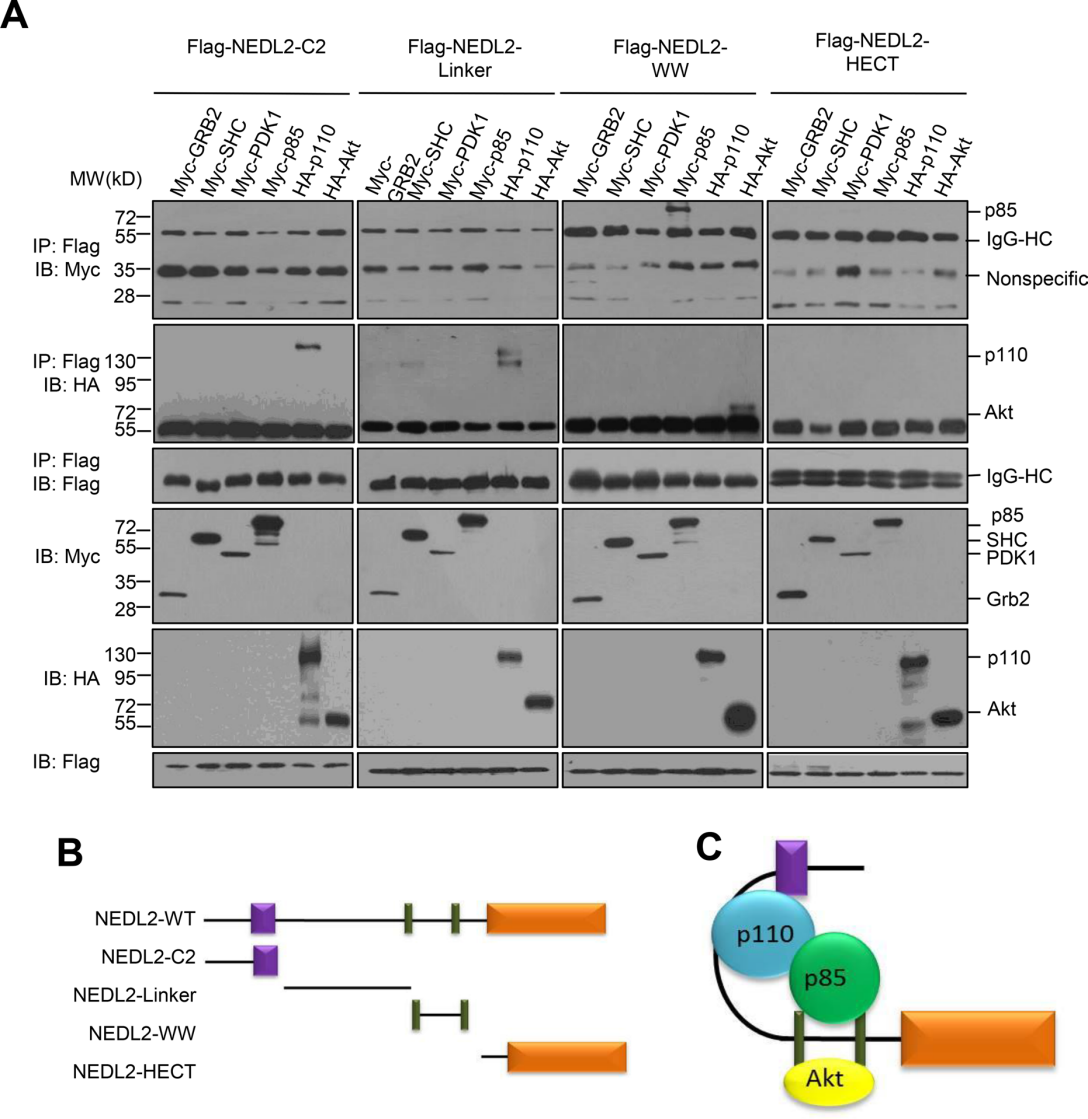
NEDL2 binds p85, p110 and Akt with different domains **A.** HEK293T cells were transfected with Flag-tagged NEDL2 constructs with Myc-SHC/Myc-Grb2/Myc-PDK1/Myc-p85/HA-p110/HA-Akt expression plasmids, Forty-eight hours after transfection, cell lysates were prepared and immunoprecipitated with anti-Flag antibody. The immunoprecipitates were analyzed by immunoblotting with the indicated antibodies. **B-C.** Schematic representation of NEDL2 constructs used in this study and the interactions of NEDL2 constructs with related proteins.

To address the the mechanism of NEDL2 in regulating the GDNF/Ret/Akt pathway, we measured the effect of NEDL2 on recruiting p85, p110 and Akt to the cell membrane, because we found that NEDL2 translocated from the cytoplasm to the plasma membrane after GDNF stimulation (Supplementary Figure S2). It has been reported that p85, p110 and Akt reside primarily in the cytosol, and these signaling molecules are recruited to the plasma membrane subsequently after activated by growth factor stimulation [6, 22]. Our results showed that more p85, p110 and Akt proteins were detected in the membrane fraction in *Nedl2^+/+^* intestine samples than that of *Nedl2^−/−^* intestine samples (Figure 6D). Furthermore, we found that NEDL2 bound to SHC, Grb2, p85, p110, PDK1, Akt but not Ret (Figure 6E). To identify the regions that are responsible for the interaction, we generated a series of NEDL2 truncated mutants, and found that the C2 domain and the C2-WW linker of NEDL2 mediated the interaction with p110, whereas the WW domains bound p85 and Akt (Figures 7A–7C). To our surprise, we did not find SHC, Grb2 and PDK1 interacted with any of the examined truncates of NEDL2 (Figure 7A). We propose that the binding of SHC, Grb2, or PDK1 to NEDL2 requires the integrity of NEDL2 whole molecule, which pattern is different from that of p85, p110, or Akt1 with NEDL2. Taken together, these results suggested that NEDL2 might work as a scaffold to integrate SHC, Grb2, PI3K, PDK1 and Akt to promote the GDNF/Ret signaling transduction.

### NEDL2 activates GDNF/Ret/Akt pathway in a Nedd8 ligase-dependent manner

**Figure 8:**
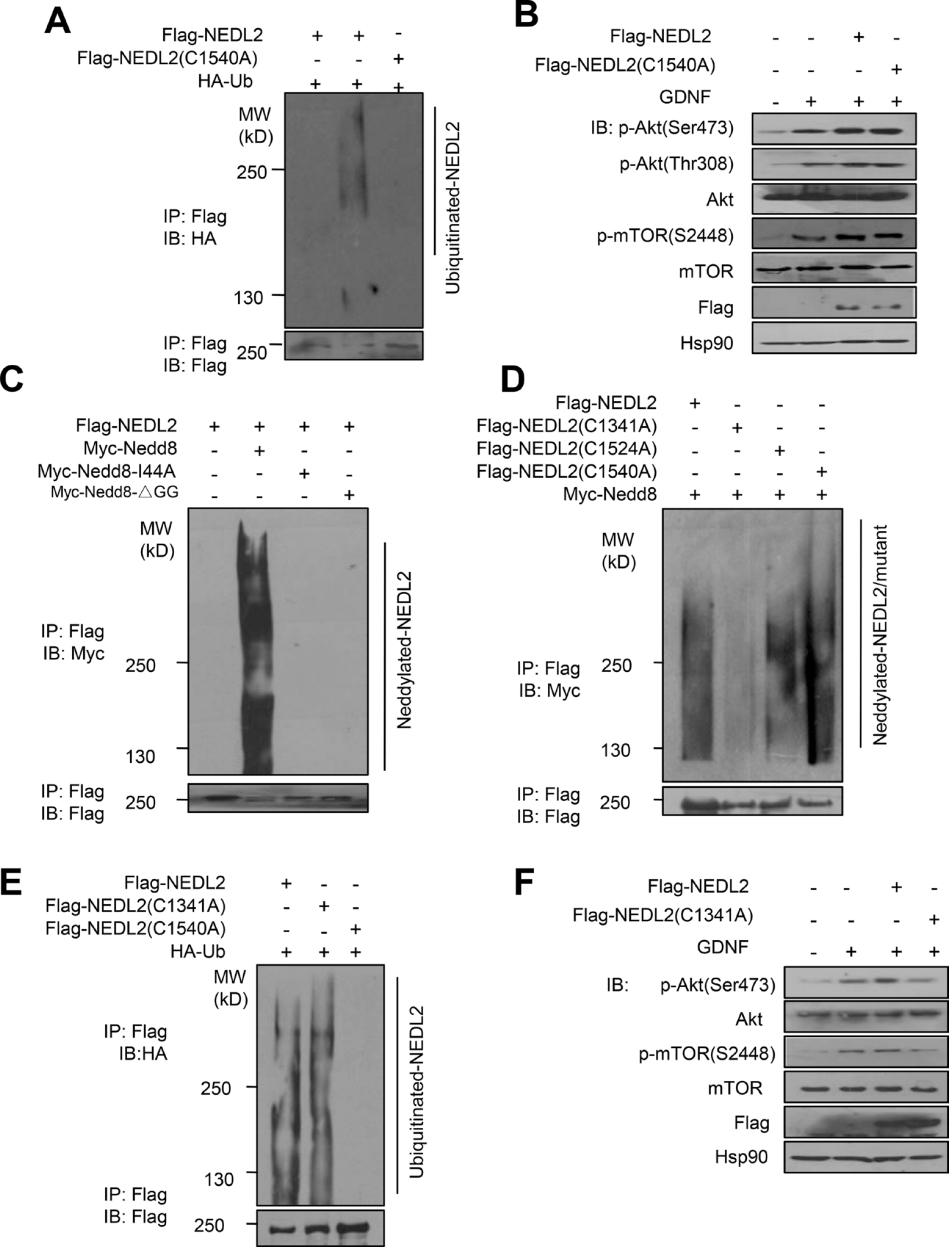
NEDL2 regulates GDNF/Ret/Akt pathway in a Nedd8 ligase-dependent manner **A-B.** NEDL2 positively regulates PI3K-Akt pathway in a ubiquitin ligase activity-independent manner. HEK293T cells were transfected with or without HA-Ub and NEDL2/NEDL2(C1540A) mutant plasmids. Ten hours before harvest, the cell were treated with MG132, and then cell lysates were prepared and immunoprecipitated with anti-Flag antibody. The immunoprecipitates were analyzed by immunoblotting with the indicated antibodies (A). MCF7 cells were transfected with or without NEDL2/NEDL2(C1540A) mutant plasmids. Cells were serum-starved overnight and either untreated or treated with GDNF (40 ng/mL, 15min); then analyzed by immunoblotting with the indicated antibodies (B). (C-F) NEDL2 positively regulates PI3K-Akt pathway in a Nedd8 ligase activity-dependent manner. **C.** HEK293T cells were transfected with Myc-Nedd8/Myc-Nedd8 I44A mutant/Myc-Nedd8ΔGG mutant and NEDL2 mutant plasmids, and then cell lysates were prepared and immunoprecipitated with anti-Flag antibody. The immunoprecipitates were analyzed by immunoblotting with the indicated antibodies. **D.** HEK293T cells were transfected with Myc-Nedd8 and the indicated NEDL2 WT and mutant plasmids, and then cell lysates were prepared and immunoprecipitated with anti-Flag antibody. The immunoprecipitates were analyzed by immunoblotting with the indicated antibodies. **E.** HEK293T cells were transfected with HA-Ub and NEDL2 plasmids, and then cell lysates were prepared and immunoprecipitated with anti-Flag antibody. The immunoprecipitates were analyzed by immunoblotting with the indicated antibodies. **F.** MCF7 cells were transfected with or without NEDL2/NEDL2(C1341A) mutant plasmids. Cells were serum-starved overnight and either untreated or treated with GDNF (40 ng/mL, 15 min) then analyzed by immunoblotting with the indicated antibodies.

NEDL2 is a typical E3 ubiquitin ligase of Nedd4 family, and members of this family has been classified into HECT-type E3s which can form thioester bond with ubiquitin [14]. To investigate whether NEDL2 regulates GDNF/Ret/Akt pathway dependent on its ubiquitin ligase activity, we first tested the C1540 residue of NEDL2, which is close to the C-terminus and functions as the ubiquitin E3 catalytic core site (Figure 8A). We used C1540A mutant to test its ability to regulate GDNF/Ret/Akt pathway. Surprisingly, our result showed that both wildtype NEDL2 and C1540A mutant could upregulate the Akt pathway stimulated by GDNF (Figure 8B). These results demonstrated that NEDL2 upregulates GDNF/Ret/Akt pathway independent of its ubiquitin catalytic activity.

On the other hand, we previously reported that Smurf1, another member of Nedd4 family, worked as a Nedd8 ligase as well as ubiquitin ligase [10]. This prompted us to hypothesize that NEDL2 might also be a Nedd8 ligase. To test this, we performed the auto-neddylation assay and found that NEDL2 could promote its self-neddylation (Figure 8C). Since there are totally three cysteine residues in the NEDL2 HECT domain, we then constructed other two mutants: C1341A and C1524A. We found that C1341A mutation abolished the auto-neddylation of NEDL2 (Figure 8D), indicating it is the neddylation catalytic core. Also surprisingly, we found that C1314A mutant did not affect the auto-ubiquitylation catalytic activity of NEDL2 (Figure 8E). In contrast, C1341A mutant could not upregulate the Akt pathway stimulated by GDNF as wild-type NEDL2 (Figure 8F). These findings suggest that NEDL2 upregulates GDNF/Ret/Akt pathway dependent on its Nedd8 ligase catalytic activity.

We hypothesize that NEDL2 activates GDNF/Ret/Akt pathway through neddylating one or more of the proteins it binds. We conducted experiments to test whether SHC, Grb2, p85, p110, PDK1 or Akt was the neddylation substrate of NEDL2. Neddylation assays in cultured cells showed that the neddylation of SHC, Grb2, p85, p110, or Akt was hardly detectable regardless the presence or absence of ectopic NEDL2 (Supplementary Figures S3A-S3D, S3F). Interestingly, the neddylation of PDK1 seemed to be readily detectable (Supplementary Figure S3E, lane 2); however, this neddylation was not enhanced by NEDL2 ovexpression (lane 3). These results suggest that these molecules might be not the neddylation substrates of NEDL2. So far, although we have attempted, we have not successfully identified a neddylation substrate of NEDL2, which needs more investigations in the future to figure out the mechanism of how NEDL2 regulates GDNF/Ret/Akt pathway.

## DISCUSSION

Recently, we have shown that NEDL2 is essential for ENS development and its deficiency resulted in newborn mice lethality within two weeks [8]. Here we show that, in addition to dilation of ENS, 38% of *Nedl2^−/−^* mutants showed unilateral or bilateral kidneys hydronephrosis. The phenotypes of *Nedl2*-deficient mice in intestinal tract and kidney resemble those of *Gdnf-, Ret*- or *Gfrα1-* deficient mice [23–25]. The current evidence based on knockout mice analysis indicated that NEDL2 is a *bona fide* positive regulator of ENS and kidney development. Moreover, as one of the RTKs, Ret transmits similar signals as the others. It is a major challenge to understand how signal specificity is achieved by these receptors [26]. It has been reported that Shp2 works as a negative regulator of EGF-dependent PI3K activation [27]. Since the expression pattern of NEDL2 is similar to that of GDNF/Ret pathway, we hypothesis that NEDL2 might be a specific positive regulator of GDNF/Ret /Akt signaling *in vivo*.

Whereas sharing high sequence similarities, it seems that NEDL2 plays a more important role in ENS and kidney development than NEDL1. *Nedl2^−/−^* mice died at perinatal stage with seriously abnormal in ENS and kidney [8], whereas, *Nedl1^−/−^* mice lived normally (Figure 2). Just like *Nedl2^−/−^* mice, *Nedl1^−/−^*;Nedl2*^−/−^* mice died at perinatal stage with low body weight, dilation of ENS and hydronephrosis (Figure 3). We have found that the NEDL1 and NEDL2 have different expression pattern, which is might only one of the reasons to explain the physiological function difference. At present, we still don't know yet that whether there is functional redundancy between these two genes in other tissues, for example the central nervous system (CNS), which shows high expression of both NEDL1 and NEDL2. More studies should to be done to reveal the functional relationship of NEDL1 and NEDL2.

Another critical finding of this study is that NEDL2 showed both ubiquitin and Nedd8 catalytic ligase activity. We have previously reported that Smurf1, another member of this family, also have both ubiquitin and Nedd8 catalytic activity [10]. Thus, we once again confirmed that HECT-type ligase can work as Nedd8 ligase. The findings on Smurf1 and NEDL2 imply that the other members of Nedd4 family might also be Nedd8 liagses. On the other hand, we demonstrated that NEDL2 regulates GDNF/Ret/Akt pathway depends on its Nedd8 ligase activity rather than ubiquitin ligase activity. To understand how NEDL2 regulates GDNF/Ret/Akt pathway, we firstly tested whether the proteins that NEDL2 interacted with were the neddylation substrates of NEDL2. However, the neddylation of SHC, Grb2, p85, p110, or Akt was hardly detectable regardless the presence or absence of ectopic NEDL2 (Supplementary Figures S3A-S3D, S3F). We cannot exclude the possibility that other protein(s) of GDNF/Ret/Akt pathway that we have not tested is the neddylation substrate of NEDL2. Interestingly, we found that the neddylation of PDK1 seemed to be readily detectable (Supplementary Figure S3E, lane 2), although this neddylation was not enhanced by NEDL2 ovexpression (lane 3). Which ligase is responsible for the PDK1 neddylation is worthy of further investigations.

Moreover, we found that SHC, Grb2 and PDK1 interacted with full-length NEDL2 but not any of the examined NEDL2 truncates (Figure 7). We speculated that the autoneddylation of NEDL2 might be required for the interaction with SHC, Grb2 and PDK1, thus explaining why the truncated mutants could not interact with SHC, Grb2 or PDK1. To verify this hypothesis, we tested the interaction between SHC, Grb2, PDK1 with NEDL2 WT or the autoneddylation-defective mutant. As shown in Supplementary Figure S4, unexpectedly, the mutant NEDL2-C1341A retained the ability to bind to SHC, Grb2 and PDK1, therefore denying the above speculation. Based on these observations, we propose that the binding of SHC, Grb2, or PDK1 to NEDL2 requires the integrity of NEDL2 whole molecule. It has been reported that self-interaction structures existed in Nedd4 family [28, 29], thus NEDL2 might also self-interact by forming intermolecular or intramolecular conformation, and the autoneddylatin of NEDL2 might contribute to the regulation of NEDL2 architecture.

In summary, the current study established the relationship between protein neddylation and the activation of GDNF/Ret/Akt pathway. These findings provide novel insight into not only the regulation of ENS and kidney development but also the function and mechanism of Nedd4 family of HECT-type E3 ligases.

## MATERIALS AND METHODS

### Ethics statement

Strain C57/BL mice mentioned in this paper were maintained in a humidity and temperature controlled, pathogen free housing units, with light-dark cycles. All animal work was approved by the Institutional Animal Care and Use Committee (IACUC) at the Beijing Institute of Radiation Medicine.

### Cell lines and culture

MCF7 and HEK293T cells were purchased from ATCC, and authenticated by STR profiling and tested for mycoplasma contamination by GENEWIZ. Cell lines were cultured in 90% DMEM+ 10% FBS, supplemented with 50 U/ml penicillin and 50 μg/ml streptomycin in a humidifid atmosphere of 5% CO_2_ at 37°C.

### Antibodies

All antibodies were purchased as follows: anti-NEDL2 (ab92711, Abcam), anti-Ret (ab134100, Abcam), anti-Neurofilament (ab50284, Abcam), anti-Akt (sc-8312, Sata Cruze), anti-pAkt (#4060, Cell Signaling), anti-S6K1 (#9202, Cell Signaling), anti-pS6K1 (#9234, Cell Signaling), anti-p85 (#4257, Cell Signaling), anti-p110 (#4249, Cell Signaling), anti-BrdU (#5259, Cell Signaling), anti-Myc (MBL), anti-Flag (MBL), anti-Hsp90 (sc-101494, Santa Cruz).

### Gene targeting of *Nedl1*

Strain C57BL/6 *Nedl1* genomic clones were used for construction of the targeting vector. Mice carrying the targeted *Nedl1* allele were bred with EIIa-Cre transgenic mice which resulted in the deletion of exon 8 of *Nedl1* genomic fragment and replaced by an *IRES-LacZ* gene. Offspring were intercrossed to generate homozygote *Nedl1^LacZ/LacZ^* (*Nedl1^−/−^*) mice. Animals were genotyped by PCR analysis. PCR primers for the wild *Nedl1* allele were (Forward:5′ –GTGCTGGAAATTGAAGTGAAGGACAA-3′) and (Reverse: 5′-ACAAACTACACAAGTATAAGAAGGGG-3′); the primers for the targeted *Nedl1* allele were (Forward: 5′-CGCTACCATTACCAGTTGGTCT-3′) and (Reverse: 5′-TCGTATGGAAGTGCAGTATG-3′).

### Histopathological analysis

Organs comprising all major organ systems were dissected from 2 months *Nedl1^−/−^* mice and littermate wildtype controls or P6 *Nedl1^−/−^*;Nedl2*^−/−^* mice and littermate *Nedl^−/−^*;Nedl2*^+/+^* controls(n≥4). Tissues were fixed overnight at 4°C in 4% paraformaldehyde in phosphate-buffered saline and processed for 3 μm thick paraffin wax sections.

### Acetylcholinesterase histochemistry

Guts of 2 months *Nedl1^−/−^* mice and littermate wildtype controls or p6 *Nedl1^−/−^*;Nedl2*^−/−^* mice and littermate *Nedl1^−/−^*;Nedl2*^+/+^* controls were dissected. Acetylcholinesterase (AChE) activity was demonstrated histochemically according to procedures as described previously [30].

### Whole-mount *in situ* hybridization

*In situ* hybridization was performed on whole embryos(E12.5) and organs(kidneys and intestinal tracts dissected from E18.5 mice), fixed in 4% paraformaldehyde in PBS overnight at 4°C, rinsed in PBS, and stored in 100% methanol at −20°C. Whole-mount *in situ* hybridization were performed as described [31]. The Probe primers for *Nedl1* were (5′-TTTACAGAGAGCCAGCGCAA-3′) and (5′-TCCAACAAGTGGTCTCGACG-3′), and the Probe primers for *Nedl2* were (5′-CCAGGGGGAGCCAATATTCC-3′) and (5′-TCATGGTACCCGCCTCTGTA-3′).

### Detection of pAkt and pErk1/2 immunohistochemistry

Immunohistochemical detection of pAkt and pErk1/2 was performed according to procedures described previously [32].

### Construction of NEDL2 shRNA expression plasmid

The oligonucleotide sequence used for NEDL2 silencing was *Bam*HI + sense + loop + antisense + terminator + EoRI, with shRNA-*NEDL2* (5′-GATCCGGGAGAAGATCCAATTTACTTCCTGTCAGAATAAATTGGATCTTCTCCCTTTTTG-3′) and (5′-AATTCAAAAAGGGAGAAGATCCAATTTATTCTGACAGGAAGTAAATTGGATCTTCTCCCG-3′). The negative control sequences were: (5′-GATCCGAGAGGGCCGGTAGTGTACTAGTTACTTCCTGTCAGATAACTAGTACACTACCGGCCCTCTCTTTTTG-3′) and (5′-AATTCAAAAAGAGAGGGCCGGTAGTGTACTAGTTATCTGACAGGAAGTAACTAGTACACTACCGGCCCTCTCG-3′).

### Membrane fractionation

Intestine tissues were dissected from P6 *Nedl2^−/−^* mice and littermate *Nedl2^+/+^* controls and membrane fractions were prepared using the ProteoJETTM Membrane Protein Extraction kit (Fermentas) according to the manufacturers' standard procedures.

### Histochemistry

Tissues were dissected in 10% formalin for 24h and processed for 3 μm paraffin wax sections. Primary antibodies used were against smooth muscle actin, Signal amplification and detection was performed using FITC-conjugated secondary antibodies according to the manufacturer's instructions. Sections were counterstained with DAPI (Sigma) to identify nuclei. Confocal microscopy was performed on a Zeiss LSM 510 Meta.

### Statistical analysis

Data were evaluated using a Student's 2-tailed t test. *p < 0.05 and **p < 0.01 was taken to be statistically significant. The error bars on graphs represent the mean ± standard deviation (SD).

## SUPPLEMENTARY TABLES AND FIGURES


