# Supplementary material for: NEDL2 regulates enteric nervous system and kidney development in its Nedd8 ligase activity-dependent manner
Source: Oncotarget. 2016 Apr 23;7(21):31440–53. doi: 10.18632/oncotarget.8951 (PMC5058769; doi:10.18632/oncotarget.8951)
Supplement: Supplementary file 1 [file oncotarget-07-31440-s001.pdf]

SUPPLEMENTARY TABLES AND FIGURES

Supplementary Table S1: Genotypes of offspring from *Nedl1*$^{+/-}$ heterozygous matings

Age, month	Genotype	No. of progeny
**12**	*Nedl1*$^{+/+}$	36
**12**	*Nedl1*$^{+/-}$	64
**12**	*Nedl1*$^{-/-}$	34

Supplementary Table S2: Genotypes of offspring from *Nedl1*$^{-/-}$*;Nedl2*$^{+/-}$ heterozygous matings

**Age, day**	***Nedl1***$^{-/-}$***;Nedl2***$^{+/+}$	***Nedl1***$^{-/-}$***;Nedl2***$^{+/-}$	***Nedl1***$^{-/-}$***;Nedl2***$^{-/-}$	**Total no.** of progeny
P0-6	21	42	16	79
P7	21	42	15	78
P8	21	42	14	77
P9	21	42	3	66
P10	21	42	0	63
Live birth	21	42	0	63

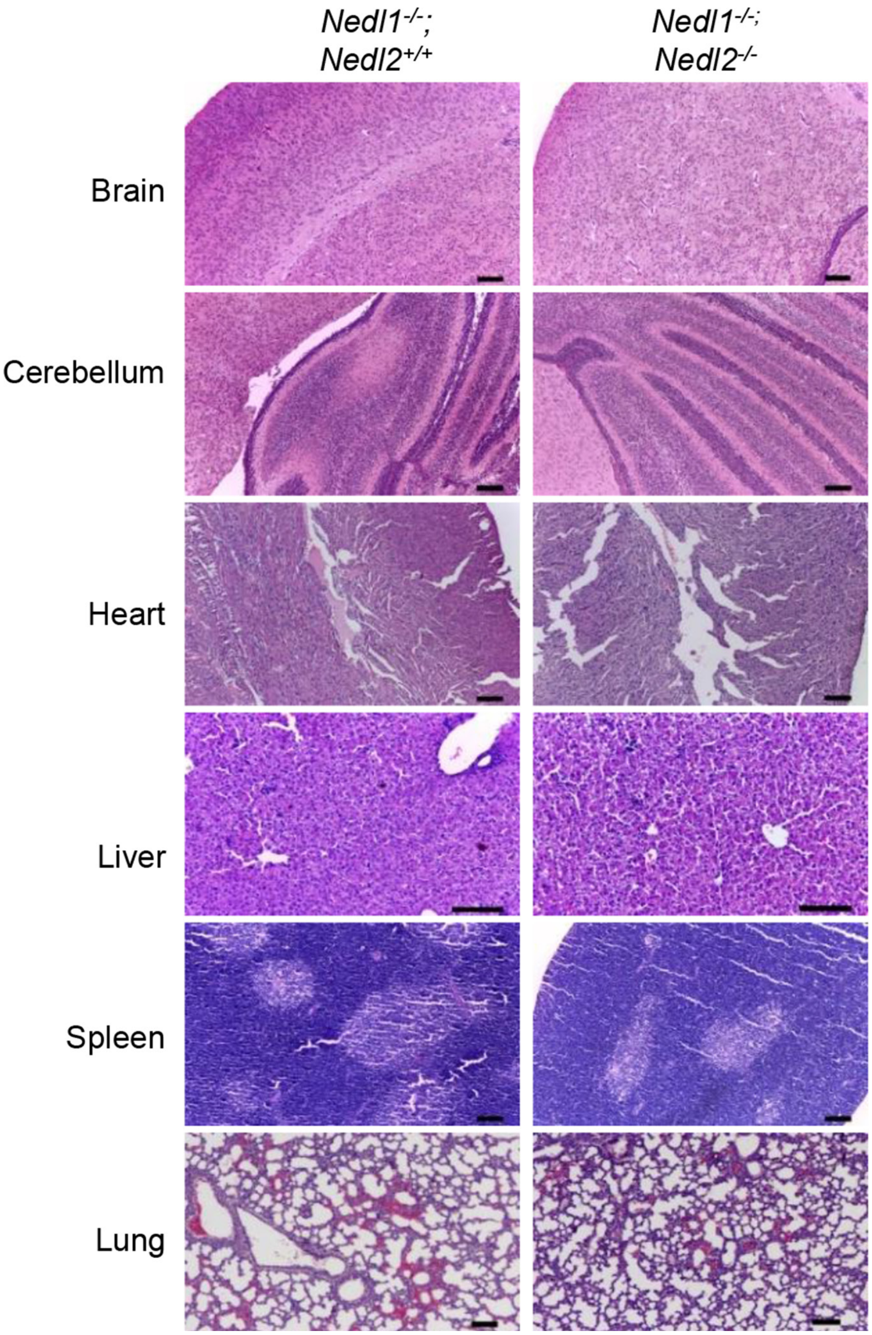

Supplementary Figure S1: Morphology analysis of *Nedl1*$^{-/-}$;*Nedl2*$^{-/-}$ mice. Analysis on HE staining of some tissue sections from P9 *Nedl1*$^{-/-}$;*Nedl2*$^{+/+}$ mice and *Nedl1*$^{-/-}$;*Nedl2*$^{-/-}$ mice. There were no obvious defects in brain, cerebellum, heart, liver, spleen and lung. Scale bar: 50 μm.

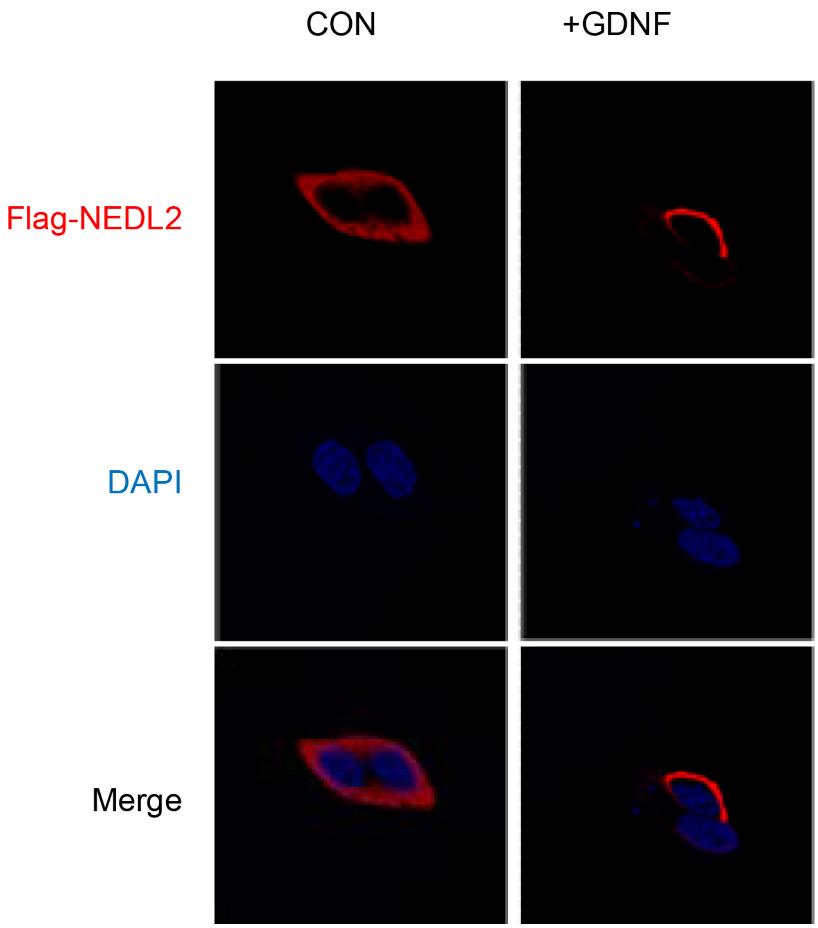

Supplementary Figure S2: GDNF induces NEDL2 to translocate from cytoplasm to plasma membrane. MCF7 cells were transfected with Flag-NEDL2. After overnight serum-starved, cells were treated with or without GDNF (40 ng/ ml, 15 min), then cells were fixed, permeabilized and immunostained with anti-Flag antibody followed by incubation with MBL anti-mouse Ig (red) secondary antibody. Nuclei were counterstained with 4,6-diamidino-2-phenylindole (DAPI) (blue).

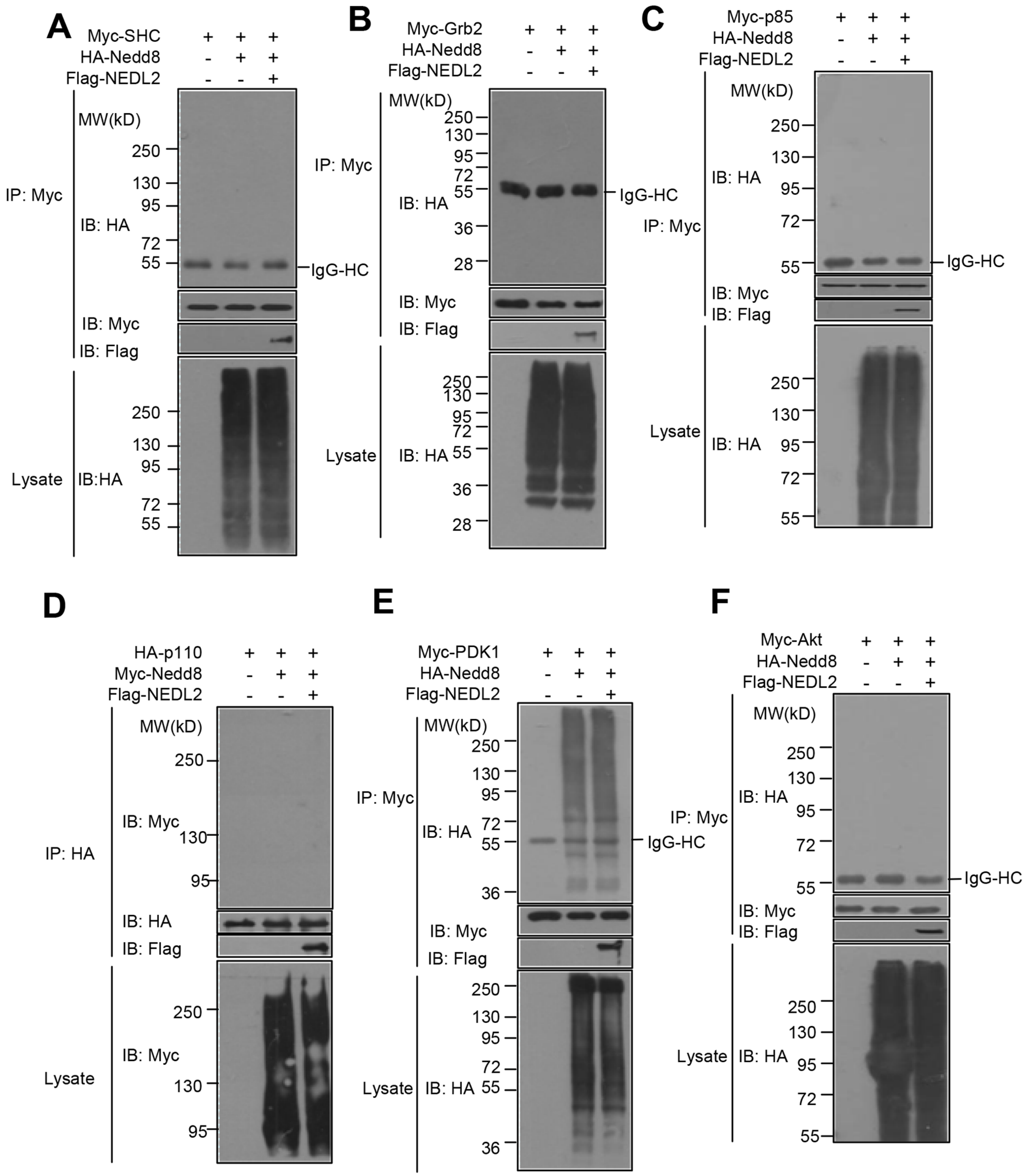

Supplementary Figure S3: SHC, Grb2, p85, p110, PDK1 and Akt are not neddylation substrates of NEDL2. HEK293T cells were transfected with HA-Nedd8, Flag-NEDL2 and Myc-SHC (A)/ Myc-Grb2(B)/Myc-p85(C)/Myc-PDK1(E)/Myc-Akt(F) plasmids as indicated, and then cell lysates were prepared and immunoprecipitated with anti-Myc antibody. The immunoprecipitates were analyzed by immunoblotting with the indicated antibodies. (D) HEK293T cells were transfected with Myc-Nedd8, HA-p110 and Flag-NEDL2 plasmids, and then cell lysates were prepared and immunoprecipitated with anti-HA antibody. The immunoprecipitates were analyzed by immunoblotting with the indicated antibodies.

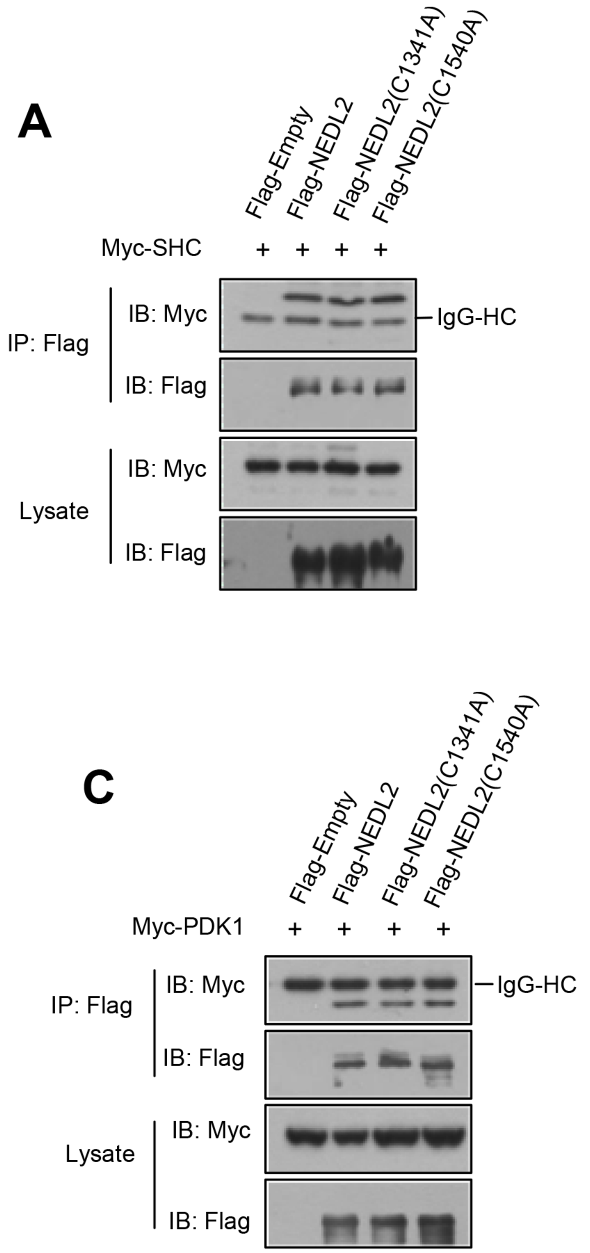

Supplementary Figure S4: SHC, Grb2 and PDK1 interact with NEDL2 independent of the Nedd8 ligase and ubiquitin ligase activity of NEDL2. HEK293T cells were separately transfected with Flag-empty, Flag-NEDL2, Flag-NEDL2(C1341A), Flag-NEDL2(C1540A) and Myc-SHC(A)/Myc-Grb2(B) /Myc-PDK1(C) plasmids, and then cell lysates were prepared and immunoprecipitated with anti-Flag antibody. The immunoprecipitates were analyzed by immunoblotting with the indicated antibodies.
